# MOTIVATIONS FOR AND EXPERIENCES OF UNDERGOING AN AMYLOID SCAN: A SYSTEMATIC REVIEW OF QUALITATIVE STUDIES

**DOI:** 10.1093/geroni/igae098.2592

**Published:** 2024-12-31

**Authors:** Elyse Couch

**Affiliations:** Brown University, Providence, Rhode Island, United States

## Abstract

Amyloid positron emission tomography (PET) scans are a tool that could improve the accuracy of dementia diagnoses. The FDA approval of new amyloid targeting monoclonal antibodies makes it increasingly likely that amyloid scans will be implemented into routine clinical care. This systematic review aimed to identify and summarize qualitative studies exploring patient and care partner experiences of undergoing an amyloid scan. Seven papers were included in this review. We identified four themes related to patient and care partner experiences of receiving an amyloid scan. 1) motivations for undergoing an amyloid scan, 2) experiences of receiving the result, 3) emotional responses to the result, and 4) actions in light of the scan result. Participants reported undergoing the scan to learn more about the patient’s condition. They described the information gained from the scan as helpful but, in some cases, struggled to recall the terminology used by the clinicians or the meaning of the scan result. Emotional responses to the scan varied depending on the scan result and those with “elevated” scan results were more likely to express feelings of distress. The most common action following the scan result was making advanced plans. Aside from determining eligibility for prescribing monoclonal antibodies, amyloid scans provide patients and their care partners with useful and actionable information. However, learning the scan result could be distressing. Future research is needed to understand how to balance the potential benefits and harms of amyloid scans in light of novel dementia treatments.

